# Barriers and facilitators to COVID-19 vaccine uptake among Australian health professional students during the pandemic: a nationwide study

**DOI:** 10.1057/s41271-023-00422-9

**Published:** 2023-06-18

**Authors:** Yingyan Chen, Roslyn Prichard, Matt Mason, Marion Tower, Peta-Anne Zimmerman, Vanessa Sparke, Janice Layh, Ahmed M. Mehdi, Frances Fengzhi Lin

**Affiliations:** 1 School of Health, University of the Sunshine Coast, Sunshine Coast, Australia; 2 School of Health and Human Sciences, Southern Cross University, Gold Coast, Australia; 3 School of Nursing and Midwifery, Griffith University, Nathan, Australia; 4 School of Nursing, Midwifery & Social Work, The University of Queensland, Brisbane, Australia; 5 School of Nursing and Midwifery, Griffith University, Southport, Australia; 6 Nursing and Midwifery, College of Healthcare Sciences, James Cook University, Cairns, Australia; 7 QCIF Bioinformatics, Queensland Cyber Infrastructure Foundation Ltd, Brisbane, Australia; 8 Sunshine Coast Health Institute, Sunshine Coast, Australia; 9 College of Nursing and Health Sciences, Flinders University, Level 1, Room N103, Sturt North Sturt Road, Bedford Park, South Australia 5042 Australia

**Keywords:** Health professional students, Risk perception, COVID-19, Vaccine hesitancy, Vaccine uptake

## Abstract

Using a cross-sectional online survey we investigated knowledge, attitudes, and risk perception about COVID-19 vaccination and identified factors influencing vaccine uptake among Australian health professional students from October 2021 to January 2022. We analysed data from 1114 health professional students from 17 Australian universities. Most participants were enrolled in nursing programs (*n* = 958, 86.8%), and 91.6% (*n* = 858) of the participants received COVID-19 vaccination. Approximately 27% believed COVID-19 was no more serious than seasonal influenza and that they had a low risk of acquiring COVID-19. Nearly 20% disagreed that COVID-19 vaccines in Australia were safe and perceived they were at higher-risk of acquiring COVID infection than the general population. Higher-risk perception viewing vaccination as their professional responsibility, and vaccine mandate strongly predicted vaccination behaviour. Participants consider COVID-19 information from health professionals, government websites, and World Health Organization as the most trusted information sources. The findings highlight that healthcare decision-makers and university administrators need to monitor students’ hesitancy with vaccination to improve students’ promotion of the vaccination to the general population.

**Supplementary Information:**

The online version contains supplementary material available at 10.1057/s41271-023-00422-9.

## Key messages

- Vaccine mandate appears to be effective in achieving a high rate of COVID-19 vaccination among health professional students.
- Nearly one in five participants perceived COVID-19 as a low-risk infection.
- Strategies, such as raising awareness about the pandemic and vaccination, are needed to address vaccine hesitancy among students.

## Introduction

As of 14 December 2022, COVID-19 had caused over 7.4 million reported deaths and 18 million estimated deaths [1]. Especially since the emergence of the highly transmissible Delta variant, Australian states and territories frequently imposed snap lockdowns. Subsequent waves, with varying in transmissibility and virulence, severely impacted delivery of healthcare services. Vaccination is widely recognized as a key strategy to manage the impacts of COVID-19, with success depending on a high level of vaccine acceptance, accessibility, and availability. The Organisation for Economic Co-operation and Development published a policy response to COVID-19, appealing for governments to collaborate to accelerate vaccination in all countries [2].

Despite much research evidence on the viability of vaccination programs in containing previous pandemics, evidence suggests that during the COVID-19 pandemic, a significant evidence-practice gap hindered vaccine uptake in many countries [3]. An online survey from 23 countries investigating vaccine hesitancy in June 2021 showed that 75.2% of 23,000 participants reported vaccine acceptance. It is imperative therefore that further strategies be considered to promote further vaccine coverage [3]. Suboptimal acceptance rates are no exception for health professional students. In a cross-sectional online survey of 2133 medical students at Tanta and Kafrelsheikh Universities of Egypt, the majority (90.5%) stated that the COVID‐19 vaccine was important, although 46% expressed vaccination hesitancy, 6% accepted the vaccine, and 6% refused it definitely [4]. A cross-sectional study of 3089 students in a French university showed that among all university students, health professional students were most likely to want to be vaccinated (75.9%); followed by midwifery, medical, and pharmacy students the most likely (61%, 59%, and 52%, respectively), and nursing students the least likely (25%) [5]. A multi-centre survey of 2249 nursing students in seven universities, including Greece, Albania, Cyprus, Spain, Italy, the Czech Republic, and Kosovo, showed that 49.7% of participants would accept vaccination if vaccines were safe and effective [6]. Commonly perceived barriers to COVID‐19 vaccination included inadequate evidence of the vaccine adverse effects, insufficient information about the vaccine itself, and concerns about vaccine safety [4, 5].

These studies provided a foundation for understanding health professional students’ vaccination uptake, but they may not reflect the Australian experience. To our knowledge, published data about COVID-19 vaccination of Australian health professional students are scarce. Identifying determinants of COVID-19 vaccination uptake in Australian health professional students could inform the development of targeted interventions to improve vaccination rates and provide insights for improving vaccine uptake in the general population. Among the five participating universities in Queensland in our study, the percentages of health professional students who had received two doses of COVID-19 vaccine, as of 20 August 2021 ranged from 20 to 43%. Only one university had achieved 73%. Hence, for the majority, rates were low compared with those of Queensland health professionals, 72% of whom had received two doses of vaccine (data reported from media source). We investigated health professional students’ knowledge, attitudes, and risk perceptions related to COVID-19 infection, risk perception about contracting the disease, and risk perception about vaccination and identified factors influencing their vaccine uptake. The study provides evidence for recommending how healthcare leaders and university administrators can improve vaccine uptake among health professional students.

## Data and methods

### Study design and theoretical framework

We designed a cross-sectional online survey by applying the Theoretical Domains Framework (TDF) to explore barriers and facilitators to vaccination uptake among health professional students to understand factors influencing their behaviour [5, 6]. TDF has been used extensively in implementation research as a “theoretical lens” to identify perceived barriers and facilitators and guide implementation of behaviour change interventions [7, 8]. Cane et al. derived this framework from 33 psychological theories and 128 theoretical constructs organised into 14 domains [7] to assess cognitive, affective, social, and environmental factors that may influence behaviour [9].

### Setting and sample

We selected participants who resided in Australia, were at least 18 years of age, and enrolled in an undergraduate, graduate, or postgraduate course in an Australian university that led to registration with the Australian Health Practitioner Regulation Agency. We excluded any registrants who studied offshore or did not reside in Australia during the study to negate factors influencing their COVID-19 vaccination uptake that might have related to factors beyond the Australian context.

### Survey tool development

To select survey questions, we consulted with senior academics involved in delivery of work-integrated learning programs (clinical placement) in Queensland, Australia. We included 89 questions in the survey in the following categories: demographic data (Sect. 1), knowledge and attitudes related to the COVID-19 pandemic, risk perception about the COVID-19 disease (Sect. 2), risk perception about COVID-19 vaccination (Sect. 3), sources of information (Sect. 4), and vaccination uptake and intention (Sect. 5). We categorized questions using 10 TDF domains [7]. A free text space was provided at the end of the survey for participants to add any additional information. Findings from the free text data will be reported in a separate paper. We piloted the draft survey with a group of selected students (*n* = 4) from two participating universities to test the electronic interface, clarity of questions, and time commitment. We incorporated minor feedback from these students and the research team (*n* = 8) into the final draft (Supplementary Material Table S1).

### Data collection procedure

We designed the survey using the Qualtrics program [10] and distributed it following ethics approvals from the researchers’ universities and gatekeeper approvals from other participating universities. Each researcher sent an email containing study information, survey link, and participant information to all health professional students at their respective universities, including five Universities (University of the Sunshine Coast, University of Queensland, Griffith University, James Cook University, and Southern Cross University), and other Australian universities through the researchers’ professional networks. The survey link remained active for two weeks for participants at each university. We sent a reminder to each participant at the end of week 1. We collected data from October 2021 to January 2022.

### Data analysis

We included a total of 1114 survey responses in the data analysis after removing 26 responses (who did not meet inclusion criteria) and another 287 (with missing data from Sect. 2 of the survey). We used descriptive statistics and presented continuous data as mean ± standard deviation (SD) or median ± interquartile range (IQR) based on normality tests. We presented categorical variables in counts and proportions (n, %). We applied Chi-square tests to investigate associations between knowledge and attitudes related to the COVID-19 pandemic, perceptions of risks related to the COVID-19 disease and its vaccination with participant health-related employment outside of their study, time progressed towards degree completion, and age.

We used multivariate logistic regression to identify predictors of vaccination uptake (received COVID-19 vaccination [yes = 1, no = 0]). We estimated odds ratios (ORs) of receiving COVID-19 vaccination using all the variables that showed significance (*p* < 0.05) in a univariate analysis and followed appropriate regression assumption tests. We used ORs and their 95% confidence intervals to measure associations between predictors and outcomes of interest. We considered a two-sided *p* < 0.05 statistically significant for all analyses.

### Ethics

The study has been approved by the University of the Sunshine Coast Human Research Ethics Committee (ethics number A211644) and ratified by four other universities.

## Results

### Participant characteristics and their vaccination uptake

**Fig. 1:**
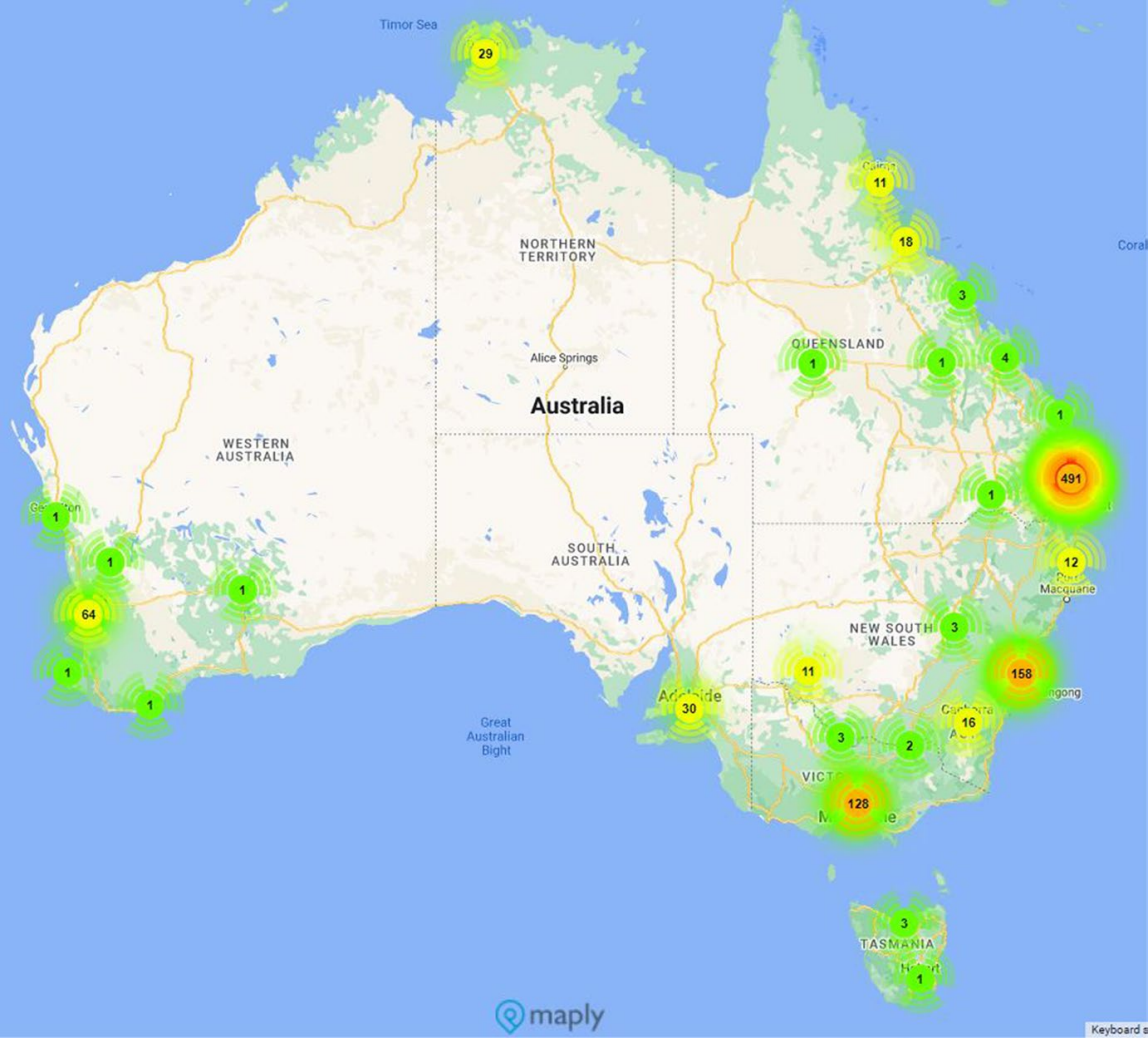
Heat map illustrating where our participants were at the time of the survey. Numbers indicate the number of participants from that geographical area. For the colors, green indicates the low numbers, yellow slightly higher, and then orange much higher with red for the highest numbers

Table 1 provides the summary of participant characteristics. Most participants were female (*n* = 958; 86.8%), with a mean age of 30 ± 10.9 years, and studied in 17 Australian universities. Participants came from 50 countries, and most resided in Queensland at the time of the survey (Fig. 1). Most participants were enrolled in nursing programs (*n* = 802; 72.2%), with the remaining in midwifery (*n* = 78; 7.0%), occupational health (*n* = 77; 6.9%), paramedicine (*n* = 54; 4.9%), psychology (*n* = 23; 2.1%), physiotherapy (*n* = 12; 1.1%), osteopathy (*n* = 11; 1.0%), dental practice (*n* = 11; 1.0%), podiatry (*n* = 2; 0.2%), pharmacy (*n* = 2; 0.2%), and others not specified (*n* = 7; 0.6%). The majority received COVID-19 vaccination (*n* = 858; 91.6%). Of those who did not, 30.3% (*n* = 23) planned to receive vaccines.

**Table 1:** Participant characteristics and their vaccination uptake and intention

Item (*n* =)^a^	*n* (%) or mean (SD)
Age (*n* = 1056)	30 (10.9)
Gender (*n* = 1104) Female	958 (86.8%)
Country born (*n* = 1114) Australia	825 (74.1%)
Speak another language (*n* = 1098) Yes	228 (20.8%)
Current program of study (*n* = 1111) Nursing	802 (72.2%)
How far toward completion (*n* = 1112)	
Less than 1 year	412 (37.1%)
1–2 years	378 (34.0%)
More than 2 years	322 (29.0%)
Plan to complete study (*n* = 1102)	
Less than 1 year	378 (34.3%)
1–2 years	484 (43.9%)
More than 2 years	240 (21.8%)
Employment outside study (*n* = 1112) Yes	915 (82.3%)
Employment outside of study – health-related (*n* = 915) Yes	541 (60.5%)
Carer responsibility outside of study (*n* = 1110) Yes	486 (43.8%)
Regular travel overseas prior to the pandemic (*n* = 1110) Yes	496 (44.7%)
Received COVID-19 vaccination (*n* = 937) Yes	858 (91.6%)
If not, plan to receive COVID-19 vaccination (*n* = 76) Yes	23 (30.3%)

^a^Due to missing values, the total numbers for many items are different

*SD* standard deviation

### Associations between knowledge, attitudes, and perceptions of risks related to the COVID-19 pandemic and COVID-19 disease with three participant characteristics

**Fig. 2:**
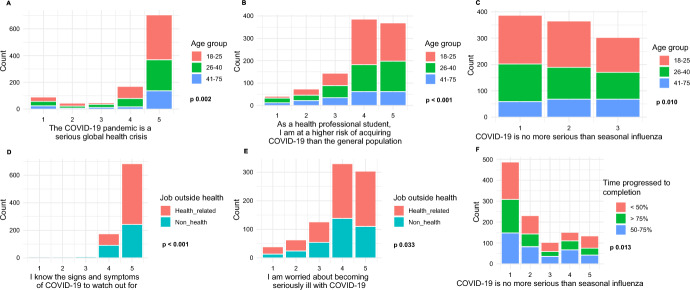
Associations between knowledge, attitudes, and risk perception about the COVID-19 pandemic/COVID-19 disease with three participant characteristics, including age, job outside health, and time progressed to degree completion. The *x*-axes are the Likert Scales with 1 strongly disagreed and 5 strongly agreed

We conducted Chi-square tests to assess associations between knowledge and attitudes related to the COVID-19 pandemic, perceptions of risks related to the COVID-19 disease with three participant characteristics: health-related employment outside their study, time progressed towards degree completion, and age (Fig. 2; Table S2). Participants who had health-related employment outside their study were more likely to know COVID-19 signs and symptoms and worry about becoming seriously ill with COVID-19 (*p* < 0.05) than those who had non-health-related employment. However, there were no statistically significant relationships between types of employment outside study and the perceived COVID-19 disease seriousness (*p* = 0.303), and the risk of acquiring COVID-19 infection (*p* = 0.493). Participants having completed 50–75% of the time needed to finish their programs and those in the 41–75 years age group (12 participants over 60 years old) were more likely to agree that COVID-19 was no more serious than seasonal influenza and that they had a lower risk of acquiring COVID-19 infection (both *p* < 0.05) than those participants who completed less than 50% or over 75% of the time needed to finish their programs and those less than 41 years old.

### Associations between knowledge, attitudes, and perception of risks related to COVID-19 vaccination with three participant characteristics

**Fig. 3:**
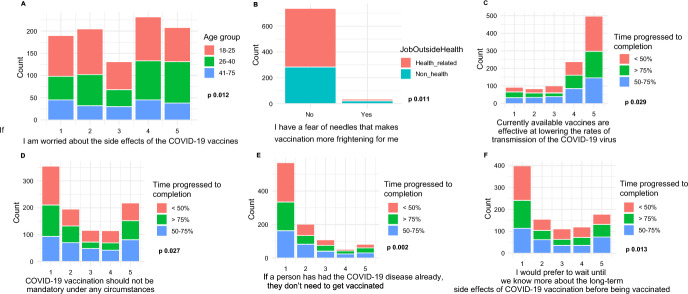
Associations between knowledge, attitudes, and risk perception about the COVID-19 vaccination with three participant characteristics, including age, job outside health, and time progressed to degree completion. The *x*-axes (except B) are the Likert Scales with 1 strongly disagreed and 5 strongly agreed

Figure 3 and Table S3 show associations between knowledge, attitudes, and perceptions of risks related to COVID-19 vaccination and participant characteristics, including health-related employment outside study, time progressed towards degree completion, and age. Participants working in a non-health-related field were more likely to fear needles, which made vaccination more frightening (*p* = 0.011), than those who worked in a health-related field. Those having reached more than 75% of time needed to complete their degrees were more likely to believe that the available vaccines were effective in lowering the rate of COVID-19 transmission (*p* = 0.029) than those who have completed less than 75%. Those in the range of 26–40 years of age were more likely to “strongly/somewhat agree” that they were worried about the vaccine side effects than those in 18–25 years and in 41–75 years groups, and those in the 18–25 years old age were more likely to fear vaccination needles (all *p* < 0.05) than the other two age groups.

Overall, more than 20% disagreed that the COVID-19 vaccines in Australia were safe. Nearly 30% “strongly/somewhat agreed” that COVID-19 vaccination should not be mandatory under any circumstances and that they would prefer to wait until they knew more about the long-term vaccine side effects before being vaccinated.

### Predictors of vaccination uptake

**Fig. 4:**
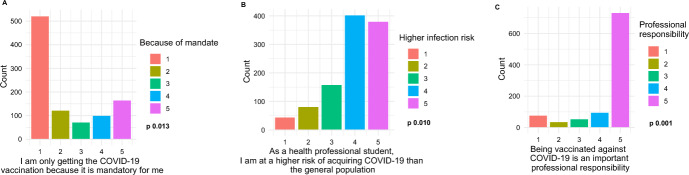
Three factors are strong predictors of vaccination uptake. The *x*-axes are the Likert Scales with 1 strongly disagreed and 5 strongly agreed

The multivariate logistic regression analyses (Fig. 4; Table S4) revealed that factors strongly predictive of vaccination uptake included: vaccine mandate, views of vaccination as an important professional responsibility, and perceptions of a higher risk of acquiring COVID-19 infection than the general population. Participants who strongly/somewhat agreed that they received COVID-19 vaccines because of the mandate (OR 328.450 [CI 11.087–9729.799], *p* < 0.001; OR 83.039 [CI 2.526–2729.900], *p* = 0.013, respectively), because COVID-19 vaccination is an important professional responsibility (OR 289.550 [CI 7.260–11,548.763], *p* = 0.003; OR 138.711 [CI 6.613–2909.534], *p* = 0.001, respectively), and because they held perceptions of higher risk of acquiring COVID-19 infection than the general population (somewhat agreed) (OR 30.643 [CI 2.246–418.125], *p* = 0.010) had higher odds of having received vaccination compared to the reference groups.

### Information sources

**Fig. 5:**
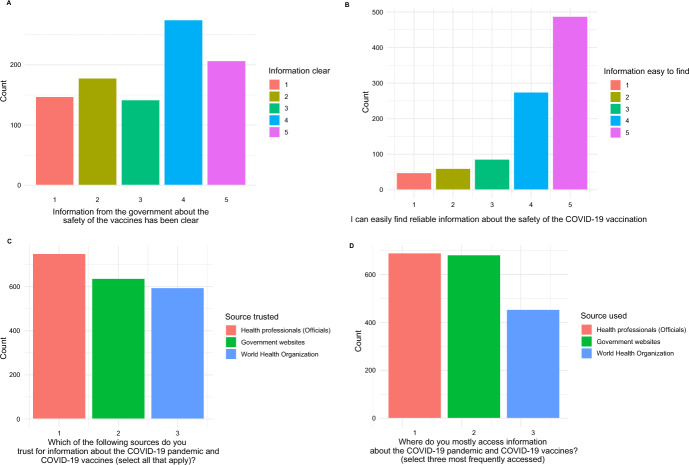
Information sources. The *x*-axes (except C and D) are the Likert Scales with 1 strongly disagreed and 5 strongly agreed. Panels C and D show that the three most trusted and used information sources about the COVID-19 pandemic and vaccines were health professionals, government websites, and World Health Organization

Participants reported that the three most trusted and used information sources about the COVID-19 pandemic and vaccines were health professionals, government websites, and World Health Organization (Fig. 5; Table S5). Of participants, 11.3% (*n* = 108) did not think finding reliable information about vaccine safety was easy, and 34.3% (*n* = 326) disagreed that the information from the government about vaccine safety was clear.

## Discussion

To our knowledge, this was the first study exploring health professional students’ COVID-19 vaccination uptake in Australia. Our study findings are particularly important because health professional students should serve as role models by participating in health promotion and health education processes and influence attitudes and health behaviour in the general population about the COVID-19 pandemic and vaccination. Indeed, among the participants, 91.6% of the participants received the vaccination, a much higher rate than health professional students in other high-income settings reported in the literature [3, 11, 12].

Doubts about the seriousness of COVID-19 disease and the perceived low risk of developing the disease can be barriers to vaccine acceptance [13] and were closely associated with time completed toward our participant attainment of the degree and age. Consistent with other studies and possibly reflecting an optimism bias [14, 15], approximately 27% of the participants in our study believed that COVID-19 disease was no more serious than seasonal influenza and that they had a lower risk of acquiring COVID-19 infection than the general population. Researchers have interpreted doubts about the seriousness of the COVID-19 disease as ‘pandemic denial’ [13, 16], and believe it links to negative attitudes and perceptions of low risk of acquiring COVID-19 infection. In our study, most participants were from Queensland, a state that had great success in controlling spread and maintaining low rates of mortality from COVID-19 at the time of the survey. Those in our study with 50–75% time progressed towards their degree completion and those of 41–75 years of age (*N* = 195) were more likely to doubt the seriousness of COVID-19 disease and hold perceptions that they had a lower risk of acquiring COVID-19 infection than the general population. Because young adults (18–29 years) had the lowest hospitalization and death rates among adult age groups [17], we expected younger participants to report lower risk perception of acquiring COVID-19 infection than the older ones. Also, we expected the general attitude of health professional students towards COVID-19 would improve in line with their progress through their courses of study due to increased knowledge. However, this was not the case. Our findings suggest that university administrators should support health professional students to understand their personal risk of COVID-19 infection and their risk of spreading COVID-19 infection to the broader community as part of their course delivery at university.

Attitudes toward vaccine safety and effectiveness may have contributed to vaccine hesitancy [13]; these were closely associated with time toward degree completion. In our study, over 20% of the participants disagreed that the COVID-19 vaccines in Australia were safe, and nearly 30% preferred to wait until they knew more about the long-term vaccine side effects before being vaccinated. This could be related to uncertainty: worries about the speed of vaccine development [5], concerns about unforeseen future effects [18], and distrust in health authorities and governments [13]. We also found that those having completed over 75% of time needed to attain their degrees were more likely to believe that the available vaccines effectively lowered the COVID-19 transmission rates due to the growing mass of vaccinated population. The findings align with previously reported literature that final-year university health students show a more positive attitude toward COVID-19 vaccination than students in earlier years of the program [19]. This fits the assumption that the attitudes of health professional students toward vaccination will improve with professional knowledge and training in their final year of study. We did not find any significant associations between employment outside study, participant age, and attitudes toward COVID-19 vaccination. Other studies have found that students employed as healthcare workers were more likely to get vaccinated [11], and health students under 25 years old held a more positive attitude toward COVID-19 vaccination than those above 25 years [18]. A qualitative study may broaden our understanding of inherent beliefs and reasoning underpinning our health professional students’ attitudes and perceptions of vaccination.

We observed three positive predictors of COVID-19 vaccination uptake including higher-risk perception of acquiring COVID infection than the general population, viewing vaccination as their professional responsibility, and vaccine mandate, with vaccine mandate as the most significant predictor. Notably, in the months prior to data collection the Queensland government mandated COVID-19 vaccination for all health professionals including health professional students. Failure to comply resulted in the termination of employment for health professionals and the withdrawal of placement for health professional students. Thus, the consequence of not following the COVID-19 vaccination mandate was significant, and likely explains why approximately 25% respondents had received vaccination against COVID-19 because of the mandate.

The proportion of students who opposed the COVID-19 vaccine mandate varied but was high worldwide, with nearly 30% of participants opposed to the mandatory COVID-19 vaccination policy in our study (Australia), 51% in Germany [20], and 71.2% in Cyprus (71.2%) [21]. In a cross-sectional survey conducted at a university in the United States, 59.1% of the students indicated that individuals should have the right to choose whether to receive the vaccine [11]. Regardless, the mandates did improve the vaccination rate; however, the hesitancy and the proportion of students opposed to the COVID-19 vaccination should not be overlooked. Future research investigating barriers and facilitators to vaccine update among students is needed to inform vaccine rollout in future pandemics.

Approximately 75% of the participants held views of vaccination as an important professional responsibility, another strong predictor. Consistent with our findings, an Australian qualitative exploration of healthcare workers reported that an expectation of their role was responsibility to receive the vaccine [22]. Similarly, studies found “collective responsibility” [5], “a community responsibility rather than a personal choice” [15], and “responsibility to receive the vaccine to protect other people from COVID-19” [11] as reasons for people to receive the vaccination. All seem equivalent to our health professional students’ view of their responsibility to the profession. Thus, instilling a sense of professional responsibility in healthcare, and education for roles in this field may improve vaccination uptake among health professional students and healthcare workers.

Another strong predictor of vaccination was the perception of a higher risk of acquiring COVID-19 infection than the general population. This is consistent with the fact that risk perception plays a significant role in vaccine uptake, a critical predictor of people’s vaccination behaviour [23]. Nursing students with perceptions of high risk of acquiring COVID-19 infection are more likely to receive vaccination than those with low-risk perception [24]. The Health Belief Model helps us understand how individual risk perception and disease seriousness can influence uptake of a recommended health behaviour [25], possibly through increasing individual knowledge, awareness, and understanding of the COVID-19 disease and its vaccine [24]. An Egyptian study, however, found that the perceived risk of acquiring COVID-19 infection did not significantly predict medical students’ intentions of getting the COVID-19 vaccine [4]. Future research should focus on potential moderating and mediating factors that influence students’ decisions about vaccine uptake while exploring associations between risk perceptions of acquiring COVID-19 infection and their intentions to receive the vaccine.

The three most trusted and used information sources about the COVID-19 pandemic and vaccines in our study were health professionals, government websites, and World Health Organization. Similarly, a cross-sectional study of 2341 students at a diverse, public university in the United States reported government and medical professionals had been the most trusted sources of information [11]. Many students in this study found information about COVID-19 vaccines difficult to understand [11]. Although our participants did not find information difficult to understand, 34.3% (*n* = 326) stated that information from the government about vaccine safety was unclear. Contrary to our findings on commonly used and trusted sources, social media has been reported as the main source of COVID-19 vaccination information in many studies, including a general population study in Cyprus (31.6%) [21], health students in Vietnam (88.5%) [26], and medical students in Pakistan (92.5%) [27]. Although we need to consider factors such as political distrust, leading to reliance on social media [21, 26, 27], this is concerning as social media gives users an outlet to express thoughts and feelings. Their anti-vaccination and vaccine hesitancy may be easily spread and discourage people from taking important health actions [13]. Governments and health professionals must deliver clear and transparent messages using layperson language, considering preferred means of access and communication for varied populations [28]. This approach may prevent people from preferring social media as a reliable source of information.

### Study limitations

Our findings represented self-reported perceptions of student who were willing to participate. Students who did not participate might have different attitudes toward vaccine hesitancy, thus we may have underestimated the true prevalence of vaccine uptake among this cohort. We received responses mainly from Queensland in Australia, limiting the generalization of findings to the whole country and other countries. As most participants were nursing students, findings may not represent all health professional students. The findings are representative of a high-income country only, with stringent border controls and no other countries neighbouring its boundaries. We did not ask the participants if they had received one or two doses of the vaccine at the time of the survey. Our survey tool was not validated, and we did not clarify if ‘acquiring’ meant ‘infection’ in one of our survey questions, which may have biased our responses. Despite these limitations, the sample size was large, and we drew the sample from multiple universities, which gives a realistic picture of the topic in Australia. The study findings provide useful insights for governments, policymakers, and university authorities responsible for developing strategies for future vaccine uptake and pandemic management.

## Conclusions

Our study shows that Australian health professional students had a high vaccination rate. Their concerns persist, however, about COVID-19 infection and disease seriousness and vaccine safety and effectiveness against acquiring infection. This implies a need for healthcare decision-makers and university administrators to invest resources to mitigate vaccine hesitancy and raise awareness about the pandemic and vaccination among university health professional students. The purpose will be to improve their willingness to recommend COVID-19 vaccination to the general population to limit ravages of COVID-19 or other pandemics.

## Supplementary Information

Below is the link to the electronic supplementary material.

Supplementary file1 (DOCX 104 kb)

## Data Availability

Data will be available upon a request.
