# Supplementary material for: Barriers and facilitators to COVID-19 vaccine uptake among Australian health professional students during the pandemic: a nationwide study
Source: J Public Health Policy. 2023 Jun 18;44(3):400–14. doi: 10.1057/s41271-023-00422-9 (PMC10485127; doi:10.1057/s41271-023-00422-9)
Supplement: Supplementary file 1 — Supplementary file1 (DOCX 104 kb) [file 41271_2023_422_MOESM1_ESM.docx]

**Journal of Public Health Policy**

Supplementary Materials

Barriers and facilitators to COVID-19 vaccine uptake among Australian health professional students during the pandemic: a nationwide study

Yingyan Chen ^1,2^, Roslyn Prichard ^1^, Matt Mason ^1^, Marion Tower ^3,4^, Peta-Anne Zimmerman ^5^, Vanessa Sparke ^6^, Janice Layh ^1^, Ahmed M. Mehdi ^7^, Frances Fengzhi Lin ^1,8,9*^

1. School of Health, University of the Sunshine Coast, Sunshine Coast, Australia
2. School of Health and Human Sciences. Southern Cross University, Gold Coast, Australia
3. School of Nursing and Midwifery, Griffith University, Nathan, Australia
4. School of Nursing, Midwifery & Social Work, The University of Queensland, Brisbane, Australia
5. School of Nursing and Midwifery, Griffith University, Southport, Australia
6. Nursing and Midwifery, College of Healthcare Sciences, James Cook University, Cairns, Australia
7. Ahmed M. Mehdi, Queensland Cyber Infrastructure Foundation Ltd, Facility for Advanced Bioinformatics, Brisbane, Australia
8. Sunshine Coast Health Institute, Sunshine Coast, Australia
9. College of Nursing and Health Sciences, Flinders University, Australia

Corresponding Author:

Frances Fengzhi Lin

frances.lin@flinders.edu.au

Level 1, Room N103, Sturt North Sturt Road, Bedford Park, Flinders University,

South Australia, 5042 Australia

Running Title:

Students’ COVID-19 vaccine uptake study

**Journal of Public Health Policy**

Supplementary Materials

Barriers and facilitators to COVID-19 vaccine uptake among Australian health professional students during the pandemic: a nationwide study

Supplementary file Table S1

**Health professional students’ knowledge, attitudes, and risk perception about COVID-19 and its vaccination question pool**

**Section 1**

Demographics

|  | **Question type and options** |
| --- | --- |
| 1. How do you describe yourself? | Drop Down   - Female or woman - Male or man - Non-Binary - Different term (Text) - Prefer not to say |
| 1. What was your age at your last birthday | Text box |
| 1. In which country were you born | Drop Down   - Australia - New Zealand - India - China - Philippines - Vietnam - Italy - South Africa - Malaysia - United Kingdom - Other (text box) |
| 1. What is your cultural background? - with which group do you most strongly identify? | Drop Down   - Australian - Australian-Aboriginal, Torres Strait Island, or South Sea Island descent - New Zealander - New Zealander Maori descent - Northwest European (British Irish, Western and Northern European) - Southern / Eastern European - Chinese - Southeast Asian (Mainland and Maritime) - Southern and Central Asian - North African / Middle Eastern (Arab, Jewish, Sudan North Africa, Middle East) - North American - South American (South and Central Americas) - Sub Saharan African (Central, West and Southeast) - Other – Text box |
| 1. Do you speak another language other than English at home? | Y/N |
| 1. What is the postcode of your current residence? | Text box |
| 1. What is your current program of study? | Drop Down   - Aboriginal and Torres Strait Islander Health Practitioners - Chinese Medicine - Chiropractic - Dental practice - Medical radiation - Medicine - Midwifery - Nursing - Occupational Health - Optometry - Osteopathy - Paramedicine - Pharmacy - Physiotherapy - Podiatry - Psychology - Other (text box) |
| 1. In which Australian institution are you currently enrolled? | Text box |
| 1. In terms of your current program of study, how far have you progressed towards completion? | Drop Down/   - Less than 50% - 50-75% - More than 75% |
| 1. When do you plan to complete your current program of study? | Drop Down   - Less than 1 year - 1-2 years - More than 2 years |
| 1. Do you have a job outside of study?    1. If yes, in which area do you mostly work | Y/N  Drop Down   - Arts and performance - Education - Healthcare Aged care - Healthcare other - Hospitality - Labourer, cleaner - Manager administrator - Sales or service worker - Trades - Other (text box) |
| 1. Do you have carer responsibilities outside of your program of study? | Y/N |
| 1. Prior to the pandemic, did you regularly travel overseas? | Y/N |

**Section 2**

Knowledge and attitudes related to the COVID-19 pandemic

| **Likert scale responses**  **1-Strongly disagree, 2-Somewhat disagree, 3-Neutral, 4-Somewhat agree, 5-Strongly agree** | | **TDF Domains** | **Barrier** | **Enabler** |
| --- | --- | --- | --- | --- |
| 1. Have you ever been diagnosed with COVID-19? 2. If “Yes,” how serious were your symptoms | Y/N Branched  Dropdown if Yes   - None or mild - Moderate - Severe |  |  |  |
| 1. Has a relative, friend, or colleague had COVID-19? 2. If “Yes,” how serious was the worst case that you are personally aware of | Y/N Branched  Dropdown if Yes   - Mild - Moderate - Severe - Death - Don’t know |  |  |  |
| 1. The COVID-19 pandemic is a serious global health crisis | 1-5 | 1-Knowledge |  | x |
| 1. COVID-19 is no more serious than the seasonal influenza | 1-5 | 1-Knowledge | x |  |
| 1. The COVID-19 pandemic has negatively impacted my life in the last 18 months | 1-5 | 6-Consequences | x | x |
| 1. The COVID-19 pandemic will negatively affect my life in the next 12 months | 1-5 | 6-Consequences | x | x |
| 1. I know the signs and symptoms of COVID-19 to watch out for | 1-5 | 1-Knowledge  2-Skills | x | x |
| 1. Mask wearing is an essential element in controlling the spread of the virus that causes COVID-19 | 1-5 | 1-Knowledge | x | x |
| 1. Physical distancing is an effective element in controlling the spread of the virus that causes COVID-19 | 1-5 | 1-Knowledge | x | x |
| 1. Hand hygiene is an effective element in controlling the spread of the virus that causes COVID-19 | 1-5 | 1-Knowledge | x | x |
| 1. I have a good knowledge of the appropriate PPE for working in a healthcare field with respect to COVID-19 | 1-5 | 1-Knowledge  2-Skills | x | x |

Risk perception related to the COVID-19 disease

| **Likert scale responses**  **1-Strongly disagree, 2-Somewhat disagree, 3-Neutral, 4-Somewhat agree, 5-Strongly agree** | |  | **Barrier** | **Enabler** |
| --- | --- | --- | --- | --- |
| 1. Have any members of your family received a COVID-19 vaccine? 2. If yes, did they experience any side effects that you know of? | Y/N Branched, Not sure  Dropdown if yes   - None or very mild - Mild - Moderate - Severe | 1-Knowledge  6-Consequences |  | x |
| 1. Have any of your friends received a COVID-19 vaccine? 2. If yes, did they experience any side effects that you know of? | Y/N Branched,  Not sure  Dropdown if yes   - None or very mild - Mild - Moderate - Severe | 1-Knowledge  6-Consequences |  | x |
| 1. As a health professional student, I am at a higher risk of acquiring COVID-19 than the general population | 1-5 | 1-Knowledge  4-Consequences |  | x |
| 1. The risks associated with getting COVID-19 disease are greater than the risks associated with getting the COVID-19 vaccination | 1-5 | 1-Knowledge  6-Consequences |  | x |
| 1. I am worried I may pass the COVID-19 infection to members of my family | 1-5 | 13-Emotion  12-Social influences |  | x |
| 1. I am not worried about becoming seriously ill with COVID-19 | 1-5 | 1-Knowledge  4-Consequences | x | x |
| 1. I am concerned about people I work with contracting COVID-19 from me | 1-5 | 4-Consequences |  | x |
| 1. I am concerned about developing long COVID-19 if I were to contract the disease | 1-5 | 4-Consequences |  | x |
| 1. I am concerned that contracting COVID-19 could negatively affect fertility | 1-5 | 6-Consequences  1-Knowledge |  | x |
| 1. I am concerned about the effects on the mother and baby of developing the COVID- 19 disease during pregnancy | 1-5 | 6-Consequences  1-Knowledge |  | x |
| 1. I have a specific medical condition that makes developing COVID-19 risky for me | 1-5 | 6-Consequences  1-Knowledge |  | x |

**Section 3**

Knowledge and attitudes related to the COVID-19 vaccination

| **Likert scale responses**  **1-Strongly disagree, 2-Somewhat disagree, 3-Neutral, 4-Somewhat agree, 5-Strongly agree** | | **TDF Domains** | **Barrier** | **Enabler** |
| --- | --- | --- | --- | --- |
| 1. Protecting my own health is an important reason for me to receive COVID-19 vaccination | 1-5 | 1-Knowledge |  | x |
| 1. Protecting my family is an important reason for me to receive COVID-19 vaccination | 1-5 | 3-Social professional role |  | x |
| 1. Protecting my community is an important reason for me to receive COVID-19 vaccination | 1-5 | 3-Social professional role |  | x |
| 1. Protecting my patients or clients is an important reason for me to receive COVID-19 vaccination | 1-5 | 3-Social professional role |  | x |
| 1. If I have had COVID-19 already, I don’t need to get vaccinated | 1-5 | 4-Beliefs about capability | x |  |
| 1. COVID-19 vaccination will help protect me from becoming seriously ill if I develop a COVID-19 viral infection | 1-5 | 6- Beliefs about consequences |  | x |
| 1. The currently available COVID-19 vaccines available in Australia are safe | 1-5 | 1-Knowledge |  | x |
| 1. Currently available vaccines are effective at lowering the rates of transmission of the COVID-19 virus | 1-5 | 1-Knowledge |  | x |
| 1. Currently available vaccines are effective at reducing hospitalisation and death for people who contract COVID-19 | 1-5 | 1-Knowledge |  | x |
| 1. I am not worried about the side effects of the COVID-19 vaccines | 1-5 | 6-Beliefs about consequences 1-Knowledge |  | x |
| 1. I actively encourage my family and friends to receive COVID-19 vaccinations | 1-5 | 14-Behavioural regulation  8-Intentions  13-Emotions |  | x |
| 1. Being vaccinated against COVID-19 is an important professional responsibility | 1-5 | 3-Social professional role |  | x |
| 1. Health workers (including students) should be vaccinated against COVID-19 to protect themselves | 1-5 | 3-Social / professional role  4-Beliefs about capability |  | x |
| 1. Health workers (including students) should be vaccinated against COVID-19 to protect others, including patients and the community | 1-5 | 3-Social / professional role  4-Beliefs about capability |  | **x** |
| 1. COVID-19 vaccination should not be mandatory under any circumstances | 1-5 | 6-Beliefs about consequences | x |  |
| 1. I am concerned that the COVID-19 vaccination could negatively affect my fertility | 1-5 | 6-Beliefs about consequences 1-Knowledge | x |  |
| 1. I am concerned that the COVID-19 vaccination could negatively affect my health | 1-5 | 6-Beliefs about consequences 1-Knowledge | x |  |
| 1. I am concerned that vaccines contain ingredients that are unacceptable for me | 1-5 | 6-Beliefs about capabilities 1-Knowledge | x |  |
| 1. I am only getting the COVID-19 vaccination because it is mandatory for me | 1-5 | 12-Social influences | x |  |
| 1. Life won't get back to normal until most people in Australia are vaccinated | 1-5 | 4-Capabilities 5-Optimism |  | x |

Risk perception related to COVID-19 vaccination

| **Likert scale responses**  **1-Strongly disagree, 2-Somewhat disagree, 3-Neutral, 4-Somewhat agree, 5-Strongly agree** | |  | **Barrier** | **Enabler** |
| --- | --- | --- | --- | --- |
| 1. Healthcare workers (including students) should receive COVID-19 vaccination | 1-5 | 1-Knowledge  6-Consequences |  | x |
| 1. Pregnant healthcare workers (including students) should be vaccinated against COVID-19 | 1-5 | 6-Consequences  1-Knowledge |  | x |
| 1. Healthcare workers (including students) in **aged care facilities** should be vaccinated for COVID-19 | 1-5 | 1-Knowledge  6-Consequences |  | x |
| 1. Healthcare workers (including students) in **acute care facilities** should be vaccinated for COVID-19 | 1-5 | 1-Knowledge  6-Consequences |  | x |
| 1. Healthcare workers (including students) in **the community** should be vaccinated for COVID-19 | 1-5 | 1-Knowledge  6-Consequences |  | x |
| 1. COVID-19 vaccination should be mandatory for healthcare workers (including students) | 1-5 | 1-Knowledge  6-Consequences |  | x |
| 1. The health risks associated with getting the Astra Zeneca vaccination is high for me | 1-5 | 6-Consequences  1-Behavioural regulation  1-Knowledge | x |  |
| 1. The health risks associated with getting the mRNA (Moderna or Pfizer) vaccination is high for me | 1-5 | 6-Consequences  1-Behavioural regulation  1-Knowledge | x |  |
| 1. I have a specific health condition that makes COVID 19 vaccination risky for me | 1-5 | 6-Consequences  1-Behavioural regulation | x |  |
| 1. I am concerned about the negative effects of vaccination for people who are breastfeeding | 1-5 | 6-Consequences  1-Behavioural regulation | x |  |
| 1. I am concerned that the COVID-19 vaccination could negatively affect fertility | 1-5 | 6-Consequences  1-Behavioural regulation | x |  |
| 1. I have allergies that make the COVID-19 vaccines unsafe for me | Y/N | 6-Consequences  1-Behavioural regulation | x |  |
| 1. I have a fear of needles that makes vaccination more frightening for me | Y/N | 1-Behavioural regulation | x |  |
| 1. The COVID-19 vaccines were developed too quickly to be safe | 1-5 | 6-Consequences  1-Behavioural regulation  1-Knowledge | x |  |
| 1. I would prefer to wait until we know more about the long term side effects of COVID-19 vaccination before being vaccinated | 1-5 | 6-Consequences  1-Behavioural regulation  1-Knowledge | x |  |
| 1. It is possible to develop the COVID-19 disease directly from the vaccination | 1-5 | 6-Consequences  1-Behavioural regulation  1-Knowledge | x |  |

**Section 4**

Sources of information

| **Likert scale responses**  **1-Strongly disagree, 2-Somewhat disagree, 3-Neutral, 4-Somewhat agree, 5-Strongly agree** | | **TDF Domain** | **Barrier** | **Enabler** |
| --- | --- | --- | --- | --- |
| 1. I consider myself to be well informed about the COVID-19 pandemic | 1-5 | 4-Beliefs about capabilities | x | x |
| 1. I consider myself to be well informed about COVID-19 vaccines | 1-5 | 4-Beliefs about capabilities | x | x |
| 1. I can easily find reliable information about the safety of the COVID-19 vaccination | 1-5 | 11-Context and resources  1-Knowledge  2-Skills | x | x |
| 1. I trust that pharmaceutical companies have been required to prove that their vaccines are safe | 1-5 | 12-Social influences |  | x |
| 1. I trust that state/territory governments are working in the best interests of my community | 1-5 | 12-Social influences |  | x |
| 1. I trust the federal government is working in the best interests of my community | 1-5 | 12-Social influences |  | x |
| 1. Media articles and shows that I see on TV and /or posts on social media **discourage** me from getting the COVID-19 vaccination | 1-5 | 12-Social influences | x |  |
| 1. Media articles and shows that I see on TV and /or posts on social media **encourage** me to have the COVID-19 vaccination | 1-5 | 12-Social influences |  | x |
| 1. Information from the government about the safety of the vaccines has been clear | 1-5 | 12-Social influences | x | x |
| 1. Information from my university about the requirement for vaccination in my course has been clear | 1-5 | 12-Social influences | x | x |
| 1. I am confident that when I talk to my primary care provider, any concerns I have about the safety of COVID-19 vaccination will be taken seriously | 1-5 | 5-Optimism 12-Social influences |  |  |
| 1. I am confident that when I talk to my university or college, any concerns I have about the safety of COVID-19 vaccination will be taken seriously | 1-5 | 5-Optimism 12-Social influences |  |  |
| 1. I am confident that when I talk to my employer, any concerns I have about the safety of COVID-19 vaccination will be taken seriously | 1-5 | 5-Optimism 12-Social influences |  |  |
| 1. Which of the following sources do you trust for information about the COVID-19 pandemic and COVID-19 vaccines (**select all that apply**)? | 1. **Official Public Australian:**  - TV-ABC/ SBS - Health professionals - Government websites - Radio/ Podcasts  1. **Australian Commercial**  - TV - News/ Magazines/Website - Radio / Podcast - Printed News magazines  1. **Social Media**  - Facebook - YouTube - Instagram - WhatsApp - TikTok - Weibo - WeChat - Other (text)  1. **Friends and family in Australia** 2. **Friends and family overseas** 3. **My Community**  - Community leader - Community Media - Religious leaders - University teachers - University peers  1. **International sources**  - World Health Organization - Overseas TV/ newspapers - Foreign websites/ - Radio overseas based  1. OTHER-text | 12-Social influences  11-Context and resources |  |  |
| 1. Where do you mostly access information about the COVID-19 pandemic and COVID-19 vaccines? **(select three most frequently accessed)** | 1. **Official Public Australian:**  - TV-ABC/ SBS - Health professionals - Government websites - Radio/ Podcasts  1. **Australian Commercial**  - TV - News/ Magazines/Website - Radio / Podcast - Printed News magazines  1. **Social Media**  - Facebook - YouTube - Instagram - WhatsApp - TikTok - Weibo - WeChat - Other (text)  1. **Friends and family in Australia** 2. **Friends and family overseas** 3. **My community**  - Community leader - Community Media - Religious leaders - University teachers - University peers  1. **International sources**  - World Health Organization - Overseas TV/ newspapers - Foreign websites/ - Radio overseas based  1. OTHER-text | 12-Social influences  11-Context and resources |  |  |

**Section 5**

Vaccination uptake and intention

| **Likert scale responses**  **1-Strongly disagree, 2-disagree, 3-Neutral, 4-Agree, 5-Strongly agree** | |  | **Barrier** | **Enabler** |
| --- | --- | --- | --- | --- |
| 1. Have you received a COVID-19 vaccination? | Y/N Branched |  | x | x |
| 1. Accessing the COVID-19 vaccination recommended for me has been difficult | 1-5 | 11-Context and resources | x |  |
| 1. Finding the time to get my COVID-19 vaccination has been difficult for me | 1-5 | 11-Context and resources | x |  |
| 1. Accessing my preferred COVID-19 vaccine has been difficult | 1-5 | 11-Context and resources | x |  |
| 1. Ethically it is important that I am vaccinated against COVID-19 | 1-5 | 3-Social/professional role and identity |  | x |
| 1. Most of my family have had, or intend to get COVID-19 vaccinations | 1-5 | 12-Social influences |  | x |
| 1. Most of my friends have had, or intend to get COVID-19 vaccinations | 1-5 | 12-Social influences |  | x |

Supplementary file Table S2: Knowledge, attitudes, and beliefs related to the pandemic, and risk perception of COVID-19 disease by three characteristics

| **Variables** | **Employment outside study** | | | **Time progressed towards completion** | | | | **Age** | | | |
| --- | --- | --- | --- | --- | --- | --- | --- | --- | --- | --- | --- |
|  | Health-related n=541 (%) | Non-health n=353 (%) | p value | < 50%  n=412 (%) | 50-75% n=378 (%) | > 75% n=322 (%) | p value | 18-25  n=494 (%) | 26-40  n=367 (%) | 41-75  n=195 (%) | p value |
| **The COVID-19 pandemic is a serious global health crisis** | | | | | | | | | | | |
| Strongly disagree | 48 (8.9) | 32 (9.1) | 0.083 | 44 (10.7) | 25 (6.6) | 30 (9.3) | 0.161 | 33 (6.7) | 33 (9.0) | 24 (12.3) | **0.002** |
| Somewhat disagree | 16 (3.0) | 21 (5.9) |  | 10 (2.4) | 19 (5.0) | 18 (5.6) |  | 22 (4.5) | 15 (4.1) | 7 (3.6) |  |
| Neither agree nor disagree | 26 (4.8) | 13 (3.7) |  | 19 (4.6) | 19 (5.0) | 11 (3.4) |  | 12 (2.4) | 23 (6.3) | 12 (6.2) |  |
| Somewhat agree | 82 (15.2) | 67 (19.0) |  | 68 (16.5) | 69 (18.3) | 46 (14.3) |  | 91 (18.4) | 63 (17.2) | 16 (8.2) |  |
| Strongly agree | 369 (68.2) | 220 (62.3) |  | 271 (65.8) | 246 (65.1) | 217 (67.4) |  | 336 (68.0) | 233 (63.5) | 136 (69.7) |  |
| **COVID-19 is no more serious than seasonal influenza** | | | | | | | | | | | |
| Strongly disagree | 252 (46.6) | 142 (40.2) | 0.303 | 179 (43.4) | 148 (39.2) | 160 (49.7) | **0.013** | 202 (40.9) | 159 (43.3) | 106 (54.4) | **0.010** |
| Somewhat disagree | 105 (19.4) | 82 (23.2) |  | 88 (21.4) | 82 (21.7) | 61 (18.9) |  | 115 (23.3) | 78 (21.3) | 24 (12.3) |  |
| Neither agree nor disagree | 46 (8.5) | 33 (9.3) |  | 44 (10.7) | 36 (9.5) | 23 (7.1) |  | 47 (9.5) | 29 (7.9) | 22 (11.3) |  |
| Somewhat agree | 66 (12.2) | 53 (15.0) |  | 41 (10.0) | 67 (17.7) | 43 (13.4) |  | 67 (13.6) | 56 (15.3) | 17 (8.7) |  |
| Strongly agree | 66 (12.2) | 43 (12.2) |  | 59 (14.3) | 42 (11.1) | 33 (10.2) |  | 61 (12.3) | 43 (11.7) | 25 (12.8) |  |
| **The COVID-19 pandemic has negatively impacted my life in the last 18 months** | | | | | | | | | | | |
| Strongly disagree | 25 (4.6) | 10 (2.8) | 0.632 | 20 (4.9) | 11 (2.9) | 9 (2.8) | 0.087 | 7 (1.4) | 18 (4.9) | 12 (6.2) | **0.006** |
| Somewhat disagree | 24 (4.4) | 17 (4.8) |  | 17 (4.1) | 12 (3.2) | 17 (5.3) |  | 13 (2.6) | 21 (5.7) | 11 (5.6) |  |
| Neither agree nor disagree | 48 (8.9) | 29 (8.2) |  | 35 (8.5) | 50 (13.2) | 23 (7.1) |  | 52 (10.5) | 33 (9.0) | 19 (9.7) |  |
| Somewhat agree | 183 (33.8) | 131 (37.1) |  | 150 (36.4) | 133 (35.2) | 110 (34.2) |  | 196 (39.7) | 122 (33.2) | 67 (34.4) |  |
| Strongly agree | 257 (47.5) | 166 (47.0) |  | 188 (45.6) | 171 (45.2) | 162 (50.3) |  | 225 (45.5) | 172 (46.9) | 86 (44.1) |  |
| **I know the signs and symptoms of COVID-19 to watch out for** | | | | | | | | | | | |
| Strongly disagree | 5 (0.9) | 3 (0.8) | **<0.001** | 4 (1.0) | 4 (1.1) | 2 (0.6) | 0.167 | 3 (0.6) | 2 (0.5) | 5 (2.6) | 0.236 |
| Somewhat disagree | 1 (0.2) | 3 (0.8) |  | 1 (0.2) | 5 (1.3) | 1 (0.3) |  | 2 (0.4) | 4 (1.1) | 1 (0.5) |  |
| Neither agree nor disagree | 4 (0.7) | 8 (2.3) |  | 6 (1.5) | 4 (1.1) | 6 (1.9) |  | 6 (1.2) | 5 (1.4) | 2 (1.0) |  |
| Somewhat agree | 84 (15.5) | 91 (25.8) |  | 97 (23.5) | 75 (19.8) | 52 (16.1) |  | 109 (22.1) | 74 (20.2) | 33 (16.9) |  |
| Strongly agree | 442 (81.7) | 242 (68.6) |  | 298 (72.3) | 284 (75.1) | 258 (80.1) |  | 366 (74.1) | 278 (75.7) | 153 (78.5) |  |
| **Mask-wearing is an essential element in controlling the spread of the virus that causes COVID-19** | | | | | | | | | | | |
| Strongly disagree | 20 (3.7) | 18 (5.1) | 0.265 | 16 (3.9) | 15 (4.0) | 16 (5.0) | 0.504 | 13 (2.6) | 15 (4.1) | 15 (7.7) | **0.014** |
| Somewhat disagree | 27 (5.0) | 25 (7.1) |  | 20 (4.9) | 24 (6.3) | 25 (7.8) |  | 27 (5.5) | 23 (6.3) | 14 (7.2) |  |
| Neither agree nor disagree | 33 (6.1) | 26 (7.4) |  | 24 (5.8) | 32 (8.5) | 17 (5.3) |  | 21 (4.3) | 31 (8.4) | 16 (8.2) |  |
| Somewhat agree | 114 (21.1) | 81 (22.9) |  | 90 (21.8) | 86 (22.8) | 71 (22.0) |  | 116 (23.5) | 84 (22.9) | 36 (18.5) |  |
| Strongly agree | 340 (62.8) | 197 (55.8) |  | 255(61.9) | 214 (56.6) | 190 (59.0) |  | 308 (62.3) | 209 (56.9) | 113 (57.9) |  |
| **Physical distancing is an effective element in controlling the spread of the virus that causes COVID-19** | | | | | | | | | | | |
| Strongly disagree | 15 (2.8) | 10 (2.8) | 0.132 | 14 (3.4) | 9 (2.4) | 7 (2.2) | 0.549 | 9 (1.8) | 10 (2.7) | 8 (4.1) | 0.353 |
| Somewhat disagree | 12 (2.2) | 17 (4.8) |  | 12 (2.9) | 14 (3.7) | 9 (2.8) |  | 17 (3.4) | 11 (3.0) | 4 (2.1) |  |
| Neither agree nor disagree | 24 (4.4) | 19 (5.4) |  | 25 (6.1) | 15 (4.0) | 19 (5.9) |  | 18(3.6) | 21 (5.7) | 15 (7.7) |  |
| Somewhat agree | 129 (23.8) | 94 (26.6) |  | 96 (23.3) | 111 (29.4) | 86 (26.7) |  | 133 (26.9) | 96 (26.2) | 50 (25.6) |  |
| Strongly agree | 354 (65.4) | 207 (58.6) |  | 257 (62.4) | 223 (59.0) | 198 (61.5) |  | 308 (62.3) | 224 (61.0) | 117 (60.0) |  |
| **Hand hygiene is an effective element in controlling the spread of the virus that causes COVID-19** | | | | | | | | | | | |
| Strongly disagree | 7 (1.3) | 4 (1.1) | 0.705 | 7 (1.3) | 2 (0.5) | 3 (1.5) | 0.688 | 5 (1.0) | 2 (0.5) | 5 (2.6) | 0.349 |
| Somewhat disagree | 1 (0.2) | 2 (0.6) |  | 1 (0.2) | 3 (0.8) | 1 (0.3) |  | 2 (0.4) | 2 (0.5) | 1 (0.5) |  |
| Neither agree nor disagree | 8 (1.5) | 6 (1.7) |  | 6 (1.5) | 8 (2.1) | 5 (1.6) |  | 5 (1.0) | 6 (1.6) | 4 (2.1) |  |
| Somewhat agree | 69 (12.8) | 53 (15.0) |  | 54 (13.1) | 54 (14.3) | 37 (11.5) |  | 56 (11.3) | 55 (15.0) | 28 (14.4) |  |
| Strongly agree | 450 (83.2) | 282 (79.9) |  | 337 (81.8) | 305 (80.7) | 273 (84.8) |  | 418 (84.6) | 297 (80.9) | 156 (80.0) |  |
| **I have a good knowledge of the appropriate PPE for working in the healthcare field with respect to COVID-19** | | | | | | | | | | | |
| Strongly disagree | 7 (1.3) | 4 (1.1) | **<0.001** | 7 (1.7) | 3 (0.8) | 2 (0.6) | **<0.001** | 3 (0.6) | 5 (1.4) | 4 (2.1) | 0.565 |
| Somewhat disagree | 2 (0.4) | 12 (3.4) |  | 9 (2.2) | 5 (1.3) | 6 (1.9) |  | 9 (1.8) | 5 (1.4) | 5 (2.6) |  |
| Neither agree nor disagree | 8 (1.5) | 17 (4.8) |  | 14 (3.4) | 14 (3.7) | 3 (0.9) |  | 13 (2.6) | 11 (3.0) | 6 (3.1) |  |
| Somewhat agree | 75 (13.9) | 99 (28.0) |  | 108 (26.2) | 78 (20.6) | 59 (18.3) |  | 119 (24.1) | 73 (19.9) | 40 (20.5) |  |
| Strongly agree | 443 (81.9) | 215 (60.9) |  | 267 (64.8) | 272 (72.0) | 249 (77.3) |  | 342 (69.2) | 268 (73.0) | 139 (71.3) |  |
| **As a health professional student, I am at a higher risk of acquiring COVID-19 than the general population** | | | | | | | | | | | |
| Strongly disagree | 26 (4.8) | 13 (3.6) | 0.493 | 13 (3.1) | 14 (37.0) | 18 (5.6) | **0.046** | 10 (2.0) | 21 (5.7) | 12 (6.2) | **<0.001** |
| Somewhat disagree | 39 (7.2) | 24 (6.7) |  | 23 (5.6) | 27 (7.1) | 32 (9.9) |  | 28 (5.7) | 24 (6.5) | 22 (11.3) |  |
| Neither agree nor disagree | 72 (13.3) | 54 (15.2) |  | 63 (15.3) | 60 (15.9) | 35 (10.9) |  | 55 (11.1) | 54 (14.7) | 35 (17.9) |  |
| Somewhat agree | 192 (35.4) | 138 (39.0) |  | 161 (39.1) | 137 (36.2) | 105 (32.6) |  | 204 (41.3) | 120 (32.7) | 62 (31.8) |  |
| Strongly agree | 194 (35.8) | 110 (31.2) |  | 134 (32.5) | 121 (32.0) | 125 (38.8) |  | 171 (34.6) | 136 (37.1) | 62 (31.8) |  |
| **The risks associated with getting COVID-19 disease are greater than the risks associated with getting the COVID-19 vaccination** | | | | | | | | | | | |
| Strongly disagree | 46 (8.5) | 34 (9.6) | 0.364 | 34 (8.3) | 36 (9.5) | 29 (9.0) | 0.038 | 36 (7.3) | 37 (10.1) | 19 (9.7) | 0.416 |
| Somewhat disagree | 17 (3.1) | 19 (5.4) |  | 12 (2.9) | 21 (5.6) | 12 (3.7) |  | 15 (3.0) | 18 (4.9) | 9 (4.6) |  |
| Neither agree nor disagree | 45 (8.3) | 31 (8.8) |  | 34 (8.3) | 42 (11.1) | 29 (9.0) |  | 42 (8.5) | 36 (9.8) | 21 (10.8) |  |
| Somewhat agree | 76 (14.0) | 40 (11.3) |  | 48 (11.7) | 64 (16.9) | 40 (12.4) |  | 64 (13.0) | 56 (15.3) | 22 (11.3) |  |
| Strongly agree | 338 (62.5) | 215 (60.9) |  | 265 (64.3) | 196 (51.9) | 205 (63.7) |  | 311 (63.0) | 207 (56.7) | 122 (62.6) |  |
| **I am worried about becoming seriously ill with COVID-19** | | | | | | | | | | | |
| Strongly disagree | 70 (12.9) | 44 (12.5) | **0.033** | 38 (9.2) | 49 (13.0) | 52 (16.1) | 0.178 | 55 (11.1) | 46 (12.5) | 23 (11.8) | 0.755 |
| Somewhat disagree | 78 (14.4) | 72 (20.4) |  | 69 (16.7) | 62 (16.4) | 51 (15.8) |  | 81 (16.4) | 55 (15.0) | 39 (20.0) |  |
| Neither agree nor disagree | 79 (14.6) | 65 (18.4) |  | 72 (17.5) | 63 (16.7) | 45 (14.0) |  | 77 (15.6) | 55 (15.0) | 38 (19.5) |  |
| Somewhat agree | 158 (29.2) | 89 (25.2) |  | 113 (27.4) | 110 (29.1) | 85 (26.4) |  | 141 (28.5) | 110 (30.0) | 48 (24.6) |  |
| Strongly agree | 135 (25.0) | 69 (19.5) |  | 100 (24.3) | 75 (19.8) | 81 (25.2) |  | 113 (22.9) | 87 (23.7) | 45 (23.1) |  |
| **I am concerned about people I work with contracting COVID-19 from me** | | | | | | | | | | | |
| Strongly disagree | 50 (9.2) | 31 (8.8) | 0.240 | 29 (7.0) | 39 (10.3) | 35 (10.9) | **0.005** | 31 (6.3) | 39 (10.6) | 22 (11.3) | 0.214 |
| Somewhat disagree | 49 (9.1) | 45 (12.7) |  | 32 (7.8) | 36 (9.5) | 49 (15.2) |  | 48 (9.7) | 39 (10.6) | 25 (12.8) |  |
| Neither agree nor disagree | 89 (16.5) | 68 (19.3) |  | 84 (20.4) | 64 (16.9) | 48 (14.9) |  | 89 (18.0) | 56 (15.3) | 40 (20.5) |  |
| Somewhat agree | 151 (27.9) | 94 (26.6) |  | 110 (26.7) | 114 (30.2) | 76 (23.6) |  | 133 (26.9) | 103 (28.1) | 50 (25.6) |  |
| Strongly agree | 181 (33.5) | 101 (28.6) |  | 138 (33.5) | 105 (27.8) | 106 (32.9) |  | 166 (33.6) | 116 (31.6) | 56 (28.7) |  |
| **I have a specific medical condition that makes developing COVID-19 risky for me** | | | | | | | | | | | |
| Strongly disagree | 271 (50.1) | 187 (53.0) | 0.187 | 217 (52.7) | 176 (46.6) | 173 (53.7) | 0.105 | 255 (51.6) | 193 (52.6) | 92 (47.2) | 0.226 |
| Somewhat disagree | 56 (10.4) | 37 (10.5) |  | 38 (9.2) | 55 (14.6) | 29 (9.0) |  | 58 (11.7) | 39 (10.6) | 20 (10.3) |  |
| Neither agree nor disagree | 85 (15.7) | 41 (11.6) |  | 47 (11.4) | 59 (15.6) | 41 (12.7) |  | 55 (11.1) | 52 (14.2) | 32 (16.4) |  |
| Somewhat agree | 48 (8.9) | 41 (11.6) |  | 43 (10.4) | 34 (9.0) | 37 (11.5) |  | 50 (10.1) | 36 (9.8) | 25 (12.8) |  |
| Strongly agree | 47 (8.7) | 22 (6.2) |  | 34 (8.3) | 26 (6.9) | 28 (8.7) |  | 30 (6.1) | 27 (7.4) | 23 (11.8) |  |
|  |  |  |  |  |  |  |  |  |  |  |  |

Supplementary file Table S3: Knowledge, attitudes, beliefs, and risk perception related to the COVID-19 vaccination by three characteristics

| **Variables** | **Employment outside study** | | | **Time progressed towards completion** | | | | **Age** | | | |
| --- | --- | --- | --- | --- | --- | --- | --- | --- | --- | --- | --- |
|  | Health-related n=541 (%) | Non-health n=353 (%) | p value | < 50%  n=412 (%) | 50-75%  n=378 (%) | > 75%  n=322 (%) | p value | 18-25  n=494 (%) | 26-40  n=367 (%) | 41-75  n=195 (%) | p value |
| **If a person has had the COVID-19 disease already, they don’t need to get vaccinated** | | | | | | | | | | | |
| Strongly disagree | 286 (52.9) | 169 (47.9) | 0.487 | 236 (57.3) | 163 (43.1) | 171 (53.1) | **0.002** | 249 (50.4) | 191 (52.0) | 104 (53.3) | 0.134 |
| Somewhat disagree | 97 (17.9) | 71 (20.1) |  | 68 (16.5) | 82 (21.7) | 52 (16.1) |  | 99 (20.0) | 61 (16.6) | 33 (16.9) |  |
| Neither agree nor disagree | 46 (8.5) | 40 (11.3) |  | 34 (8.3) | 40 (10.6) | 35 (10.9) |  | 43 (8.7) | 37 (10.1) | 26 (13.3) |  |
| Somewhat agree | 26 (4.8) | 17 (4.8) |  | 10 (2.4) | 24 (6.3) | 18 (5.6) |  | 16 (3.2) | 25 (6.8) | 10 (5.1) |  |
| Strongly agree | 38 (7.0) | 23 (6.5) |  | 22 (5.3) | 30 (7.9) | 30 (9.3) |  | 26 (5.3) | 30 (8.2) | 17 (8.7) |  |
| **COVID-19 vaccination will help protect me from becoming seriously ill if I develop a COVID-19 infection** | | | | | | | | | | | |
| Strongly disagree | 27 (5.0) | 20 (5.7) | 0.874 | 19 (4.6) | 21 (5.6) | 22 (6.8) | 0.272 | 19 (3.8) | 26 (7.1) | 12 (6.2) | 0.233 |
| Somewhat disagree | 20 (3.7) | 14 (4.0) |  | 11 (2.7) | 14 (3.7) | 16 (5.0) |  | 14 (2.8) | 17 (4.6) | 7 (3.6) |  |
| Neither agree nor disagree | 35 (6.5) | 17 (4.8) |  | 22 (5.3) | 28 (7.4) | 18 (5.6) |  | 21 (4.3) | 27 (7.4) | 16 (8.2) |  |
| Somewhat agree | 101 (18.7) | 66 (18.7) |  | 79 (19.2) | 81 (21.4) | 63 (19.6) |  | 93 (18.8) | 69 (18.8) | 42 (21.5) |  |
| Strongly agree | 310 (57.3) | 203 (57.5) |  | 248 (60.2) | 195 (51.6) | 187 (58.1) |  | 286 (57.9) | 205 (55.9) | 113 (57.9) |  |
| **The currently available COVID-19 vaccines available in Australia are safe** | | | | | | | | | | | |
| Strongly disagree | 38 (7.0) | 32 (9.1) | 0.660 | 28 (6.8) | 31 (8.2) | 31 (9.6) | **0.048** | 35 (7.1) | 32 (8.7) | 17 (8.7) | 0.308 |
| Somewhat disagree | 25 (4.6) | 17 (4.8) |  | 14 (3.4) | 24 (6.3) | 20 (6.2) |  | 16 (3.2) | 25 (6.8) | 14 (7.2) |  |
| Neither agree nor disagree | 49 (9.1) | 33 (9.3) |  | 30 (7.3) | 47 (12.4) | 32 (9.9) |  | 40 (8.1) | 38 (10.4) | 23 (11.8) |  |
| Somewhat agree | 133 (24.6) | 74 (21.0) |  | 99 (24.0) | 90 (23.8) | 72 (22.4) |  | 113 (22.9) | 92 (25.1) | 46 (23.6) |  |
| Strongly agree | 247 (45.7) | 164 (46.5) |  | 199 (48.3) | 146 (38.6) | 151 (46.9) |  | 228 (46.2) | 157 (42.8) | 90 (46.2) |  |
| **Currently available vaccines are effective at lowering the rates of transmission of the COVID-19 virus** | | | | | | | | | | | |
| Strongly disagree | 38 (7.0) | 30 (8.5) | 0.360 | 27 (6.6) | 33 (8.7) | 33 (10.2) | **0.029** | 33 (6.7) | 34 (9.3) | 16 (8.2) | 0.700 |
| Somewhat disagree | 46 (8.5) | 24 (6.8) |  | 24 (5.8) | 35 (9.3) | 25 (7.8) |  | 31 (6.3) | 29 (7.9) | 21 (10.8) |  |
| Neither agree nor disagree | 44 (8.1) | 36 (10.2) |  | 41 (10.0) | 40 (10.6) | 20 (6.2) |  | 41 (8.3) | 35 (9.5) | 20 (10.3) |  |
| Somewhat agree | 122 (22.6) | 65 (18.4) |  | 77 (18.7) | 85 (22.5) | 76 (23.6) |  | 107 (21.7) | 82 (22.3) | 38 (19.5) |  |
| Strongly agree | 242 (44.7) | 165 (46.7) |  | 201 (48.8) | 146 (38.6) | 151 (46.9) |  | 221 (44.7) | 163 (44.4) | 95 (48.7) |  |
| **Currently available vaccines are effective at reducing hospitalisation and death for people who contract COVID-19 disease** | | | | | | | | | | | |
| Strongly disagree | 22 (4.1) | 17 (4.8) | 0.576 | 19 (4.6) | 14 (3.7) | 19 (5.9) | 0.112 | 17 (3.4) | 24 (6.5) | 10 (5.1) | 0.350 |
| Somewhat disagree | 15 (2.8) | 13 (3.7) |  | 9 (2.2) | 20 (5.3) | 11 (3.4) |  | 10 (2.0) | 15 (4.1) | 9 (4.6) |  |
| Neither agree nor disagree | 40 (7.4) | 31 (8.8) |  | 27 (6.6) | 33 (8.7) | 27 (8.4) |  | 35 (7.1) | 28 (7.6) | 19 (9.7) |  |
| Somewhat agree | 107 (19.8) | 57 (16.1) |  | 75 (18.2) | 79 (20.9) | 54 (16.8) |  | 87 (17.6) | 69 (18.8) | 41 (21.0) |  |
| Strongly agree | 307 (56.7) | 201 (56.9) |  | 240 (58.3) | 191 (50.5) | 194 (60.2) |  | 283 (57.3) | 206 (56.1) | 111 (56.9) |  |
| **I am worried about the side effects of the COVID-19 vaccines** | | | | | | | | | | | |
| Strongly disagree | 101 (18.7) | 62 (17.6) | 0.284 | 82 (19.9) | 55 (14.6) | 62 (19.3) | 0.136 | 92 (18.6) | 53 (14.4) | 45 (23.1) | **0.012** |
| Somewhat disagree | 102 (18.9) | 66 (18.7) |  | 83 (20.1) | 62 (16.4) | 66 (20.5) |  | 103 (20.9) | 70 (19.1) | 32 (16.4) |  |
| Neither agree nor disagree | 63 (11.6) | 50 (14.2) |  | 51 (12.4) | 45 (11.9) | 39 (12.1) |  | 63 (12.8) | 38 (10.4) | 30 (15.4) |  |
| Somewhat agree | 129 (23.8) | 66 (18.7) |  | 86 (20.9) | 96 (25.4) | 64 (19.9) |  | 99 (20.0) | 88 (24.0) | 45 (23.1) |  |
| Strongly agree | 97 (17.9) | 75 (21.2) |  | 68 (16.5) | 80 (21.2) | 75 (23.3) |  | 77 (15.6) | 93 (25.3) | 38 (19.5) |  |
| **Being vaccinated against COVID-19 is an important professional responsibility** | | | | | | | | | | | |
| Strongly disagree | 28 (5.2) | 33 (9.3) | 0.124 | 20 (4.9) | 32 (8.5) | 27 (8.4) | **0.027** | 29 (5.9) | 29 (7.9) | 15 (7.7) | 0.093 |
| Somewhat disagree | 18 (3.3) | 10 (2.8) |  | 7 (1.7) | 16 (4.2) | 14 (4.3) |  | 13 (2.6) | 15 (4.1) | 8 (4.1) |  |
| Neither agree nor disagree | 30 (5.5) | 13 (3.7) |  | 17 (4.1) | 24 (6.3) | 14 (4.3) |  | 13 (2.6) | 22 (6.0) | 12 (6.2) |  |
| Somewhat agree | 45 (8.3) | 29 (8.2) |  | 33 (8.0) | 39 (10.3) | 25 (7.8) |  | 38 (7.7) | 42 (11.4) | 13 (6.7) |  |
| Strongly agree | 361 (66.7) | 230 (65.2) |  | 287 (69.7) | 223 (59.0) | 222 (68.9) |  | 330 (66.8) | 233 (63.5) | 140 (71.8) |  |
| **COVID-19 vaccination should not be mandatory under any circumstances** | | | | | | | | | | | |
| Strongly disagree | 180 (33.3) | 109 (30.9) | 0.246 | 145 (35.2) | 93 (24.6) | 117 (36.3) | **0.006** | 143 (28.9) | 123 (33.5) | 81 (41.5) | **0.010** |
| Somewhat disagree | 95 (17.6) | 61 (17.3) |  | 63 (15.3) | 70 (18.5) | 62 (19.3) |  | 96 (19.4) | 56 (15.3) | 31 (15.9) |  |
| Neither agree nor disagree | 54 (10.0) | 37 (10.5) |  | 44 (10.7) | 48 (12.7) | 24 (7.5) |  | 56 (11.3) | 31 (8.4) | 23 (11.8) |  |
| Somewhat agree | 60 (11.1) | 29 (8.2) |  | 46 (11.2) | 42 (11.1) | 27 (8.4) |  | 52 (10.5) | 38 (10.4) | 18 (9.2) |  |
| Strongly agree | 92 (17.0) | 79 (22.4) |  | 66 (16.0) | 81 (21.4) | 71 (22.0) |  | 76 (15.4) | 93 (25.3) | 35 (17.9) |  |
| **I am concerned that vaccines contain ingredients that are unacceptable for me** | | | | | | | | | | | |
| Strongly disagree | 223 (41.2) | 153 (43.3) | 0.447 | 177 (43.0) | 137 (36.2) | 144 (44.7) | 0.509 | 220 (44.5) | 141 (38.4) | 81 (41.5) | **0.004** |
| Somewhat disagree | 66 (12.2) | 42 (11.9) |  | 50 (12.1) | 45 (11.9) | 45 (14.0) |  | 65 (13.2) | 48 (13.1) | 19 (9.7) |  |
| Neither agree nor disagree | 90 (16.6) | 50 (14.2) |  | 67 (16.3) | 70 (18.5) | 48 (14.9) |  | 63 (12.8) | 67 (18.3) | 46 (23.6) |  |
| Somewhat agree | 46 (8.5) | 23 (6.5) |  | 29 (7.0) | 33 (8.7) | 21 (6.5) |  | 31 (6.3) | 27 (7.4) | 20 (10.3) |  |
| Strongly agree | 51 (9.4) | 43 (12.2) |  | 39 (9.5) | 42 (11.1) | 37 (11.5) |  | 38 (7.7) | 51 (13.9) | 19 (9.7) |  |
| **I am only getting the COVID-19 vaccination because it is mandatory for me** | | | | | | | | | | | |
| Strongly disagree | 257 (47.5) | 161 (45.6) | 0.781 | 203 (49.3) | 147 (38.9) | 172 (53.4) | **0.006** | 239 (48.4) | 166 (45.2) | 102 (52.3) | 0.158 |
| Somewhat disagree | 66 (12.2) | 41 (11.6) |  | 52 (12.6) | 42 (11.1) | 29 (9.0) |  | 54 (10.9) | 38 (10.4) | 21 (10.8) |  |
| Neither agree nor disagree | 32 (5.9) | 27 (7.6) |  | 29 (7.0) | 27 (7.1) | 17 (5.3) |  | 30 (6.1) | 22 (6.0) | 16 (8.2) |  |
| Somewhat agree | 43 (7.9) | 33 (9.3) |  | 32 (7.8) | 39 (10.3) | 30 (9.3) |  | 31 (6.3) | 44 (12.0) | 20 (10.3) |  |
| Strongly agree | 77 (14.2) | 48 (13.6) |  | 46 (11.2) | 72 (19.0) | 46 (14.3) |  | 62 (12.6) | 64 (17.4) | 27 (13.8) |  |
| **Life won't get back to normal until most people in Australia are vaccinated** | | | | | | | | | | | |
| Strongly disagree | 41 (7.6) | 30 (8.5) | 0.956 | 27 (6.6) | 32 (8.5) | 33 (10.2) | 0.474 | 28 (5.7) | 36 (9.8) | 20 (10.3) | **0.006** |
| Somewhat disagree | 29 (5.4) | 20 (5.7) |  | 22 (5.3) | 28 (7.4) | 18 (5.6) |  | 29 (5.9) | 28 (7.6) | 10 (5.1) |  |
| Neither agree nor disagree | 48 (8.9) | 27 (7.6) |  | 37 (9.0) | 33 (8.7) | 26 (8.1) |  | 28 (5.7) | 43 (11.7) | 19 (9.7) |  |
| Somewhat agree | 118 (21.8) | 78 (22.1) |  | 88 (21.4) | 88 (23.3) | 69 (21.4) |  | 125 (25.3) | 74 (20.2) | 38 (19.5) |  |
| Strongly agree | 241 (44.5) | 156 (44.2) |  | 188 (45.6) | 147 (38.9) | 150 (46.6) |  | 207 (41.9) | 154 (42.0) | 100 (51.3) |  |
| **The health risks associated with getting the Astra Zeneca vaccination are high for me** | | | | | | | | | | | |
| Strongly disagree | 128 (23.7) | 78 (22.1) | 0.120 | 97 (23.5) | 77 (20.4) | 72 (22.4) | 0.639 | 103 (20.9) | 78 (21.3) | 55 (28.2) | **0.035** |
| Somewhat disagree | 66 (12.2) | 49 (13.9) |  | 59 (14.3) | 47 (12.4) | 42 (13.0) |  | 75 (15.2) | 48 (13.1) | 18 (9.2) |  |
| Neither agree nor disagree | 131 (24.2) | 64 (18.1) |  | 83 (20.1) | 94 (24.9) | 68 (21.1) |  | 102 (20.6) | 90 (24.5) | 48 (24.6) |  |
| Somewhat agree | 79 (14.6) | 68 (19.2) |  | 64 (15.5) | 61 (16.1) | 55 (17.1) |  | 80 (16.2) | 61 (16.6) | 27 (13.8) |  |
| Strongly agree | 65 (12.0) | 46 (13.0) |  | 51 (12.4) | 46 (12.2) | 52 (16.1) |  | 47 (9.5) | 54 (14.7) | 36 (18.5) |  |
| **The health risks associated with getting the mRNA (Moderna or Pfizer) vaccination are high for me** | | | | | | | | | | | |
| Strongly disagree | 189 (34.9) | 115 (32.6) | 0.201 | 151 (36.7) | 114 (30.2) | 117 (36.3) | **0.020** | 165 (33.4) | 124 (33.8) | 79 (40.5) | 0.066 |
| Somewhat disagree | 92 (17.0) | 75 (21.1) |  | 85 (20.6) | 64 (16.9) | 55 (17.1) |  | 99 (20.0) | 70 (19.1) | 26 (13.3) |  |
| Neither agree nor disagree | 117 (21.6) | 60 (17.0) |  | 70 (17.0) | 91 (24.1) | 63 (19.6) |  | 91 (18.4) | 73 (19.9) | 51 (26.2) |  |
| Somewhat agree | 32 (5.9) | 22 (6.2) |  | 17 (4.1) | 30 (7.9) | 19 (5.9) |  | 23 (4.7) | 27 (7.4) | 10 (5.1) |  |
| Strongly agree | 39 (7.2) | 33 (9.3) |  | 31 (7.5) | 26 (6.9) | 35 (10.9) |  | 29 (5.9) | 37 (10.1) | 18 (9.2) |  |
| **I have a specific health condition that makes COVID-19 vaccination risky for me** | | | | | | | | | | | |
| Strongly disagree | 302 (55.8) | 201 (56.9) | 0.906 | 243 (59.0) | 199 (52.6) | 183 (56.8) | 0.408 | 283 (57.3) | 214 (58.3) | 103 (52.8) | **0.018** |
| Somewhat disagree | 55 (10.2) | 33 (9.3) |  | 34 (8.3) | 46 (12.2) | 31 (9.6) |  | 49 (9.9) | 35 (9.5) | 22 (11.3) |  |
| Neither agree nor disagree | 69 (12.8) | 43 (12.2) |  | 48 (11.7) | 49 (13.0) | 39 (12.1) |  | 46 (9.3) | 46 (12.5) | 34 (17.4) |  |
| Somewhat agree | 29 (5.4) | 16 (4.5) |  | 18 (4.4) | 18 (4.8) | 21 (6.5) |  | 22 (4.5) | 20 (5.4) | 13 (6.7) |  |
| Strongly agree | 14 (2.6) | 12 (3.4) |  | 11 (2.7) | 13 (3.4) | 15 (4.7) |  | 7 (1.4) | 16 (4.4) | 12 (6.2) |  |
| **I have allergies that make the COVID-19 vaccines unsafe for me** | | | | | | | | | | | |
| Yes | 13 (2.4) | 6 (1.7) | 0.474 | 5 (1.2) | 12 (3.2) | 9 (2.8) | 0.162 | 8 (1.6) | 8 (2.2) | 8 (4.1) | 0.234 |
| **I have a fear of needles that makes vaccination more frightening for me** | | | | | | | | | | | |
| Yes | 14 (2.6) | 21 (5.9) | **0.011** | 20 (4.9) | 17 (4.5) | 10 (3.1) | 0.394 | 29 (5.9) | 15 (4.1) | 3 (1.5) | **0.016** |
| **The COVID-19 vaccines were developed too quickly to be safe** | | | | | | | | | | | |
| Strongly disagree | 166 (30.7) | 126 (35.7) | 0.103 | 140 (34.0) | 104 (27.5) | 111 (34.5) | 0.072 | 164 (33.2) | 119 (32.4) | 60 (30.8) | 0.128 |
| Somewhat disagree | 101 (18.7) | 55 (15.6) |  | 72 (17.5) | 60 (15.9) | 62 (19.3) |  | 91 (18.4) | 54 (14.7) | 36 (18.5) |  |
| Neither agree nor disagree | 79 (14.6) | 35 (9.9) |  | 50 (12.1) | 55 (14.6) | 37 (11.5) |  | 58 (11.7) | 49 (13.4) | 30 (15.4) |  |
| Somewhat agree | 67 (12.4) | 43 (12.2) |  | 49 (11.9) | 62 (16.4) | 33 (10.2) |  | 50 (10.1) | 58 (15.8) | 29 (14.9) |  |
| Strongly agree | 54 (10.0) | 44 (12.5) |  | 41 (10.0) | 41 (10.8) | 46 (14.3) |  | 43 (8.7) | 49 (13.4) | 27 (13.8) |  |
| **I would prefer to wait until we know more about the long-term side effects of COVID-19 vaccination before being vaccinated** | | | | | | | | | | | |
| Strongly disagree | 203 (37.5) | 123 (34.8) | 0.392 | 158 (38.3) | 114 (30.2) | 127 (39.4) | **0.013** | 180 (36.4) | 121 (33.0) | 80 (41.0) | 0.359 |
| Somewhat disagree | 71 (13.1) | 53 (15.0) |  | 51 (12.4) | 62 (16.4) | 42 (13.0) |  | 69 (14.0) | 53 (14.4) | 26 (13.3) |  |
| Neither agree nor disagree | 54 (10.0) | 32 (9.1) |  | 48 (11.7) | 36 (9.5) | 27 (8.4) |  | 44 (8.9) | 39 (10.6) | 21 (10.8) |  |
| Somewhat agree | 62 (11.5) | 32 (9.1) |  | 48 (11.7) | 36 (9.5) | 36 (11.2) |  | 50 (10.1) | 48 (13.1) | 18 (9.2) |  |
| Strongly agree | 77 (14.2) | 63 (17.8) |  | 47 (11.4) | 74 (19.6) | 57 (17.7) |  | 63 (12.8) | 68 (18.5) | 37 (19.0) |  |
| **It is possible to develop the COVID-19 disease directly from the vaccines** | | | | | | | | | | | |
| Yes | 33 (6.1) | 25 (7.1) | 0.540 | 33 (8.0) | 26 (6.9) | 16 (5.0) | 0.184 | 30 (6.1) | 25 (6.8) | 15 (7.7) | 0.940 |

Supplementary file Table S4: Multivariate logistic regression model for COVID-19 vaccination uptake among health students

| **Variables** | **COVID-19 vaccination uptake OR (95% CI) (N=937)** | **p value** |
| --- | --- | --- |
| **Age (years)** |  |  |
| 18-25 | 1 (ref) |  |
| 26-40 | 0.329 (0.084-1.285) | 0.110 |
| 41-75 | 2.342 (0.316-17.374) | 0.405 |
| **Employment outside study – health-related** | 0.308 (0.083-1.148) | 0.079 |
| **The COVID-19 pandemic is a serious global health crisis** | | |
| Strongly disagree | 1 (ref) |  |
| Somewhat disagree | 0.824 (0.067-10.097) | 0.880 |
| Neither agree nor disagree | 0.075 (0.004-1.332) | 0.078 |
| Somewhat agree | 0.188 (0.014-2.483) | 0.205 |
| Strongly agree | 0.297 (0.013-6.515) | 0.441 |
| **COVID-19 is no more serious than the seasonal influenza** | | |
| Strongly disagree | 1 (ref) |  |
| Somewhat disagree | 0.220 (0.017-2.796) | 0.243 |
| Neither agree nor disagree | 1.331 (0.102-17.418) | 0.828 |
| Somewhat agree | 2.715 (0.197-37.518) | 0.456 |
| Strongly agree | 0.825 (0.074-9.130) | 0.875 |
| **Mask-wearing is an essential element in controlling the spread of the virus that causes COVID-19** | | |
| Strongly disagree | 1 (ref) |  |
| Somewhat disagree | 5.477 (0.527-56.980) | 0.155 |
| Neither agree nor disagree | 14.174 (0.527-380.871) | 0.114 |
| Somewhat agree | 9.394 (0.672-131.332) | 0.096 |
| Strongly agree | 5.131 (0.315-83.585) | 0.251 |
| **As a health professional student, I am at a higher risk of acquiring COVID-19 than the general population** | | |
| Strongly disagree | 1 (ref) |  |
| Somewhat disagree | 1.039 (0.096-11.186) | 0.975 |
| Neither agree nor disagree | 9.759 (0.727-130.965) | 0.086 |
| Somewhat agree | 30.643 (2.246-418.125) | **0.010** |
| Strongly agree | 4.950 (0.385-63.638) | 0.220 |
| **The risks associated with getting COVID-19 disease are greater than the risks associated with getting the COVID-19 vaccination** | | |
| Strongly disagree | 1 (ref) |  |
| Somewhat disagree | 3.716 (0.274-50.481) | 0.324 |
| Neither agree nor disagree | 1.664 (0.165-16.816) | 0.666 |
| Somewhat agree | 6.040 (0.425-85.818) | 0.184 |
| Strongly agree | 1.633 (0.070-37.996) | 0.760 |
| **I am worried I may pass the COVID-19 infection to members of my family** | | |
| Strongly disagree | 1 (ref) | 0.582 |
| Somewhat disagree | 0.754 (0.082-6.950) | 0.803 |
| Neither agree nor disagree | 0.461 (0.062-3.411) | 0.448 |
| Somewhat agree | 0.395 (0.051-3.034) | 0.372 |
| Strongly agree | 2.003 (0.110-36.522) | 0.639 |
| **COVID-19 vaccination should not be mandatory under any circumstances** | | |
| Strongly disagree | 1 (ref) |  |
| Somewhat disagree | 0.000 (0.000-0.035) | **0.001** |
| Neither agree nor disagree | 0.015 (0.000-0.776) | **0.037** |
| Somewhat agree | 0.016 (0.000-1.243) | 0.063 |
| Strongly agree | 0.005 (0.000-0.229) | **0.006** |
| **Being vaccinated against COVID-19 is an important professional responsibility** | | |
| Strongly disagree | 1 (ref) |  |
| Somewhat disagree | 6.771 (0.784-58.476) | 0.082 |
| Neither agree nor disagree | 9.358 (1.323-66.186) | **0.025** |
| Somewhat agree | 138.711 (6.613-2909.534) | **0.001** |
| Strongly agree | 289.550 (7.260-11548.763) | **0.003** |
| **I am only getting the COVID-19 vaccination because it is mandatory for me** | | |
| Strongly disagree | 1 (ref) |  |
| Somewhat disagree | 309497041.4 (0.000) | 0.995 |
| Neither agree nor disagree | 2.128 (0.113-40.174) | 0.614 |
| Somewhat agree | 83.039 (2.526-2729.900) | **0.013** |
| Strongly agree | 328.450 (11.087-9729.799) | **<0.001** |
| **The COVID-19 vaccines were developed too quickly to be safe** | | |
| Strongly disagree | 1 (ref) |  |
| Somewhat disagree | 0.404 (0.008-21.234) | 0.654 |
| Neither agree nor disagree | 0.012 (0.000-0.567 | **0.024** |
| Somewhat agree | 0.026 (0.001-0.839) | **0.039** |
| Strongly agree | 0.024 (0.001-0.782) | **0.036** |
| **I would prefer to wait until we know more about the long-term side effects of COVID-19 vaccination before being vaccinated** | | |
| Strongly disagree | 1 (ref) |  |
| Somewhat disagree | 122845382.7 (0.000) | 0.995 |
| Neither agree nor disagree | 0.643 (0.019-21.556) | 0.806 |
| Somewhat agree | 0.095 (0.004-2.320) | 0.149 |
| Strongly agree | 0.013 (0.000-0.359) | **0.010** |

Note: OR=odds ratio; CI=confidence interval.

Supplementary file Table S5: Source of information

| **Statements** | **Scale** | **n (%)** |
| --- | --- | --- |
| I consider myself to be well informed about the COVID-19 pandemic (n=956) | Strongly disagree | 7 (0.7%) |
|  | Somewhat disagree | 35 (3.7%) |
|  | Neither agree nor disagree | 83 (8.7%) |
|  | Somewhat agree | 432 (45.2%) |
|  | Strongly agree | 399 (41.7%) |
| I can easily find reliable information about the safety of the COVID-19 vaccination (n=957) | Strongly disagree | 48 (5.0%) |
|  | Somewhat disagree | 60 (6.3%) |
|  | Neither agree nor disagree | 86 (9.0%) |
|  | Somewhat agree | 275 (28.7%) |
|  | Strongly agree | 488 (51.0%) |
| I trust that pharmaceutical companies have been required to prove that their vaccines are safe (n=956) | Strongly disagree | 105 (11.0%) |
|  | Somewhat disagree | 83 (8.7%) |
|  | Neither agree nor disagree | 77 (8.1%) |
|  | Somewhat agree | 258 (27.0%) |
|  | Strongly agree | 433 (45.3%) |
| I trust that state/territory governments are working in the best interests of my community (n=956) | Strongly disagree | 115 (12.0%) |
|  | Somewhat disagree | 99 (10.4%) |
|  | Neither agree nor disagree | 85 (8.9%) |
|  | Somewhat agree | 271 (28.3%) |
|  | Strongly agree | 386 (40.4%) |
| I trust the federal government is working in the best interests of my community (n=956) | Strongly disagree | 135 (14.1%) |
|  | Somewhat disagree | 134 (14.0%) |
|  | Neither agree nor disagree | 106 (11.1%) |
|  | Somewhat agree | 276 (28.9%) |
|  | Strongly agree | 305 (31.9%) |
| Media articles and shows that I see on TV and /or posts on social media **discourage** me from getting the COVID-19 vaccination (n=951) | Strongly disagree | 353 (37.1%) |
|  | Somewhat disagree | 183 (19.2%) |
|  | Neither agree nor disagree | 206 (21.7%) |
|  | Somewhat agree | 146 (15.4%) |
|  | Strongly agree | 63 (6.6%) |
| Media articles and shows that I see on TV and /or posts on social media **encourage** me to have the COVID-19 vaccination (n=951) | Strongly disagree | 96 (10.1%) |
|  | Somewhat disagree | 81 (8.5%) |
|  | Neither agree nor disagree | 230 (24.2%) |
|  | Somewhat agree | 301 (31.7%) |
|  | Strongly agree | 243 (25.6%) |
| Information from the government about the safety of the vaccines has been clear (n=950) | Strongly disagree | 148 (15.6%) |
|  | Somewhat disagree | 178 (18.7%) |
|  | Neither agree nor disagree | 142 (14.9%) |
|  | Somewhat agree | 275 (28.9%) |
|  | Strongly agree | 207 (21.8%) |
| Information from my place of study about the requirement for vaccination has been clear (n=951) | Strongly disagree | 50 (5.3%) |
|  | Somewhat disagree | 59 (6.2%) |
|  | Neither agree nor disagree | 85 (8.9%) |
|  | Somewhat agree | 252 (26.5%) |
|  | Strongly agree | 505 (53.1%) |
| I am confident that when I talk to my primary care provider, any concerns I have about the safety of COVID-19 vaccination will be taken seriously (n=945) | Strongly disagree | 76 (8.0%) |
|  | Somewhat disagree | 87 (9.2%) |
|  | Neither agree nor disagree | 113 (12.0%) |
|  | Somewhat agree | 211 (22.3%) |
|  | Strongly agree | 458 (48.5%) |
| I am confident that when I talk to my place of study, any concerns I have about the safety of COVID-19 vaccination will be taken seriously (n=945) | Strongly disagree | 105 (11.1%) |
|  | Somewhat disagree | 100 (10.6%) |
|  | Neither agree nor disagree | 159 (16.8%) |
|  | Somewhat agree | 218 (23.1%) |
|  | Strongly agree | 363 (38.4%) |
| Which of the following sources do you trust for information about the COVID-19 pandemic and COVID-19 vaccines (**select all that apply**)? (n=3,660) | Health professionals (Officials) | 750 |
|  | Government websites | 637 |
|  | World Health Organization | 595 |
|  | ABC/ SBS TV | 314 |
|  | University or college teachers | 303 |
|  | ABC radio or podcasts | 189 |
|  | Friends and family in Australia | 127 |
|  | University or college peers | 87 |
|  | Friends and family overseas | 78 |
|  | Others | 127 |
| Where do you mostly access information about the COVID-19 pandemic and COVID-19 vaccines? (select three most frequently accessed) (n=2,804) | Health professionals (Officials) | 690 |
|  | Government websites | 682 |
|  | World Health Organization | 454 |
|  | ABC/ SBS TV | 273 |
|  | University or college teachers | 113 |
|  | Friends and family in Australia | 75 |
|  | ABC radio or podcasts | 56 |
|  | Other | 99 |

*Due to missing values, the total numbers for many items are different.
